# Evaluation of the Therapeutic Potential of Natural Peptides Against Hemagglutinin–Esterase of Bovine Coronavirus Based on Molecular Dynamics Simulation Studies

**DOI:** 10.1155/vmi/2269384

**Published:** 2026-09-15

**Authors:** Mohammed Adnan Hashim Al-battat, Marzieh Gharouni, Mohammadreza Nassiri, Ali Javadmanesh, Ahmad Asoodeh, Reza Tohidi

**Affiliations:** ^1^ Department of Animal Science, Faculty of Agriculture, Ferdowsi University of Mashhad, Mashhad, Iran, um.ac.ir; ^2^ Metabolic Syndrome Research Center, Mashhad University of Medical Sciences, Mashhad, Iran, mums.ac.ir; ^3^ The Research Institute of Biotechnology, Ferdowsi University of Mashhad, Mashhad, Iran, um.ac.ir; ^4^ Department of Chemistry, Faculty of Science, Ferdowsi University of Mashhad, Mashhad, Iran, um.ac.ir; ^5^ Department of Animal Science, Faculty of Agriculture, University of Torbat-e Jam, Torbat-e Jam, Iran

**Keywords:** bovine coronavirus, hemagglutinin–esterase (HE), molecular docking, molecular dynamics simulation, peptide, protein–peptide interaction

## Abstract

The hemagglutinin–esterase (HE) protein of bovine coronavirus is required for virus attachment and entry into host cells. It is thus an attractive target for the development of peptide‐based antiviral agents. In the present study, molecular docking was performed to identify bioactive peptides with high binding potential toward the HE protein, followed by molecular dynamics (MD) simulations. A total of 16 bioactive peptides were initially evaluated by molecular docking, and 3 peptides with favorable interactions at the HE protein functional site were subsequently subjected to MD simulations. After the simulation run, among the investigated peptides, Peptide 16 exhibited the most favorable interaction with the HE protein. Structural interaction analysis revealed the formation of two salt bridges, two hydrogen bonds, and forty hydrophobic interactions at the binding interface. Furthermore, the averaged MD analyses demonstrated that the HE protein–Peptide 16 (bLFcin *f*) complex remained structurally stable throughout the simulations. These findings suggest that Peptide 16 is a promising candidate for further experimental evaluation as a peptide inhibitor targeting the HE protein of BCoV.

## 1. Introduction

Infectious diseases and pandemics pose severe public health challenges. Over the past 2 decades, several coronavirus epidemics have been reported in humans and livestock [1]. Coronaviruses are classified into four genera based on their genomic structure and phylogenetic relationships: *Alphacoronavirus*, *Betacoronavirus*, *Gammacoronavirus*, and *Deltacoronavirus*. Although the *Alphacoronaviruses* and *Betacoronaviruses* primarily infect humans and other mammals, the *Gammacoronaviruses* and *Deltacoronaviruses* can infect a broad range of hosts, including avian species [2].

The bovine coronavirus (BCoV) is a pathogen of significant economic importance, responsible for enteric and respiratory infections in cattle worldwide [3]. BCoV is now recognized to be involved in at least three distinct clinical syndromes: (1) neonatal calf diarrhea with high mortality rates; (2) winter dysentery, characterized by hemorrhagic diarrhea in adult cattle; and (3) respiratory infections affecting all age groups [4]. Respiratory infections can further contribute to the bovine respiratory disease complex (BRDC), a polymicrobial syndrome and one of the most economically important infectious diseases in cattle [5, 6]. It initiates infection through the spike (S) glycoprotein. The S1 subunit mediates attachment to host cell receptors, primarily O‐acetylated sialic acids, whereas the S2 subunit promotes fusion of the viral and host membranes. Upon fusion, the viral positive‐sense RNA genome is released into the cytoplasm, where translation by the replicase complex (nsps 1–16) facilitates genome replication and transcription of subgenomic mRNAs [6]. A unique feature of BCoV and other *Embecoviruses* (a subgenus of coronaviruses in the genus *Betacoronavirus*) is the presence of the hemagglutinin–esterase (HE) protein, which functions as both a lectin and a receptor‐destroying enzyme (RDE) [7].

The HE protein of BCoV is a multifunctional Type I transmembrane glycoprotein encoded by the HE gene that plays critical roles in both receptor binding and receptor destruction. The mature HE protein comprises 424 amino acids and has an unglycosylated molecular mass of approximately 47.7 kDa [8]. Structurally, it includes a short N‐terminal signal peptide (residues 1–17), a receptor‐binding lectin domain (∼18–340), an esterase catalytic domain (∼40–150) containing a conserved Ser–His–Asp catalytic triad, a transmembrane anchor (∼388–410), and a cytoplasmic tail (∼411–424) [9]. The lectin domain allows initial reversible binding to 9‐O‐acetylated sialic acid‐containing glycoproteins or glycolipids on the host cell surface, enhancing viral attachment in coordination with the spike protein. The esterase domain then hydrolyzes acetyl groups from sialic acids during viral entry or egress, promoting virion release, preventing reinfection of infected cells, and limiting virion aggregation by removing residual receptors [7]. Crystallographic studies of the HE ectodomain (residues 19–388) have revealed that HE forms homodimers and shares both topological and functional homology with the influenza C virus HEF protein [7]. This dual functionality contributes to viral dissemination across the respiratory tract’s mucosal layer and may aid immune evasion. Furthermore, recent evidence indicates that the HE gene undergoes site‐specific mutations that affect receptor affinity and antigenic profile, suggesting ongoing adaptive evolution in both domesticated and feral cattle populations.

The BCoV genome is a single‐stranded, positive‐sense RNA approximately 31 kb in length [10]. The virus encodes five structural proteins: HE, spike (*S*), envelope (*E*), membrane (*M*), and nucleocapsid (*N*), along with several accessory proteins, including ns2, ns4.9, ns4.8, ns12.7, and protein I [3]. Like other RNA viruses, coronaviruses exhibit high mutation rates and frequent recombination events, enabling rapid adaptation to new ecological niches [11, 12]. The HE protein may contribute to the expansion of the BCoV host range by increasing the number or diversity of susceptible cell types [3, 4].

The success of antiviral therapies depends on multiple factors, including viral mutation rates, viral population genetic diversity, transmission routes, replication efficiency, and viral persistence within the host. Viruses with high replication and mutation rates can rapidly develop resistance to antiviral agents. Consequently, researchers continually seek compounds with high efficacy and minimal side effects for the management of such viruses. Unlike small‐molecule drugs, antiviral peptides (AVPs) can interfere directly with viral entry, replication, or assembly by mimicking host cell proteins or disrupting viral envelope integrity. Their ability to target protein–protein interactions, such as those between viral surface proteins and host receptors, makes them particularly effective against viruses like HIV, influenza, and coronaviruses. Moreover, peptide‐based drugs can be rapidly synthesized and modified to enhance stability and bioavailability, making them adaptable to evolving viral strains. Several peptides, including enfuvirtide (an HIV fusion inhibitor) and EK1 (a pan‐coronavirus fusion inhibitor), have demonstrated potent antiviral effects in both preclinical and clinical settings [13–15]. Given the increasing incidence of viral outbreaks and the limitations of traditional antiviral agents, peptide therapeutics constitute a valuable platform for future antiviral drug development.

Despite challenges such as enzymatic degradation, advances in computational modeling, particularly molecular docking and molecular dynamics (MD) simulations, have accelerated peptide‐based drug design by enabling efficient *in silico* screening and optimization of candidate molecules [16–18]. A notable domain within structural bioinformatics is molecular modeling and MD simulations, which have evolved significantly since the 1970s. MD simulations are widely used in biological systems, particularly in drug and vaccine design and optimization [19].

For BCoV, therapeutic strategies targeting the sugar‐recognition domain or hydrolase activity of the HE enzyme represent a promising avenue. Therefore, this study aimed to identify peptide candidates capable of disrupting the function of the BCoV HE enzyme as a potential antiviral treatment.

## 2. Materials and Methods

### 2.1. Preparation of Protein and Peptide Structures for In Silico Study

The three‐dimensional structure file of the bovine HE protein was retrieved from the RCSB Protein Data Bank (https://www.rcsb.org/) using the PDB ID 3CL4. Missing residues in the protein structure were modeled with MODELLER Version 10.2 to generate a complete, accurate structure suitable for further computational analysis. The crystal structure exhibited six glycosylation sites at N54, N89, N236, N301, N316, and N358, which were conserved in the modeled HE structure.

The peptides investigated in this study were selected based on prior studies demonstrating their diverse therapeutic properties, including antimicrobial, immunomodulatory, and other biological activities. Sequence sources and references for each peptide are provided in Table 1. First, the properties of these peptides were analyzed using a set of specialized web‐based bioinformatic servers:

**TABLE 1:** Amino acid sequences, sources, and corresponding references for the peptides evaluated in this study.

Number	Sequence	Peptide source	References
1	KAEDTQTLPFR	Quail	Unpublished data
2	FESAFNTGATNR	Lysozyme	[20]
3	NTDGSTDYGILQINSR	Lysozyme	[21]
4	KLKNFAKGVAQSLLNKASCKLSGQC	Frog	[22]
5	FLKPLFNAALKLLP	Frog	[23]
6	SSMKLSFRARAYGFRGPGPQL	Peptide bank	[24]
7	TSAKFTEEKLPNYR	Ostrich albumin	Unpublished data
8	ELQER	Ostrich egg	[25]
9	IPALLKR	Ostrich egg	Unpublished data
10	ELQACQQVMDR	Ostrich egg	Unpublished data
11	AQQLAAQLPAMCR	Ostrich egg	[25]
12	LEGGDALSASQ	Ostrich egg	[25]
13	FLPVLAGVLSRA	Ostrich egg	[23]
14	LLGTILGLLKGL	Ostrich egg	Unpublished data
15	GLFDIIKKIAESF	Frog	Unpublished data
16	RRWQWRMKKLG^1^	Peptide Bank	[26]

ToxinPred (https://webs.iiitd.edu.in/raghava/toxinpred/algo.php) for toxicity prediction; HemoPI (https://webs.iiitd.edu.in/raghava/hemopi/) for hemolytic activity assessment; ProInflam (http://metabiosys.iiserb.ac.in/proinflam/) for the prediction of proinflammatory potential; Predicting Antigenic Peptides (http://imed.med.ucm.es/Tools/antigenic.pl) for antigenicity prediction; and PEPlife (http://crdd.osdd.net/raghava/peplife/) to evaluate the half‐life of peptides. Eleven peptides (Peptides 1, 2, 6, 7, 8, 9, 10, 11, 12, 13, and 16) passed these filters and were selected for assessment of peptide–HE interaction as shown in Table 2. The three‐dimensional structures of these peptides were not available in the database, and their structures were predicted using the PEP‐FOLD3 web server (https://mobyle.rpbs.univ-paris-diderot.fr/cgi-bin/portal.py#forms::PEP-FOLD3). Second, MD simulations were performed to assess the structural stability of the peptides over a 50 ns timescale.

**TABLE 2:** Predicted properties of the peptides analyzed in this study, including toxicity, physicochemical characteristics, inflammatory potential, half‐life, and antigenicity.

Number	Sequence	Toxin Pred	HemoPred	Proinflam	Instability index	Hydrophobicity Hydropathicity /Hydrophilicity	Half‐life	Charge	PI	Predicted antigenic Peptides
1	KAEDTQTLPFR Mol wt 1305.60	Nontoxin	Nonhemolytic	Negative	41.10	−0.36 −1.23 0.60	3 min (yeast, in vivo)	0.00	6.07	0 antigenic

2	FESAFNTGATNR Mol wt 1314.53	Nontoxin	Nonhemolytic	Negative	−8.53	−0.20 −0.70 −0.01	3 min (yeast, in vivo)	0.00	6.36	0 antigenic

3	NTDGSTDYGILQINSR Mol wt 1754.08	Nontoxin	Nonhemolytic	Negative	37.91	−0.23 −0.89 0.11	3 min (yeast, in vivo)	−1.00	4.21	1 antigenic

4	KLKNFAKGVAQSLLNKASCKLSGQC Mol wt 2637.53	Nontoxin	Nonhemolytic	Negative	27.08	−0.18 −0.16 0.08	3 min (yeast, in vivo)	5.00	9.91	1 antigenic

5	FLKPLFNAALKLLP Mol wt 1585.22	Nontoxin	Nonhemolytic	Negative	0.10	−0.54 0.98 −0.63	3 min (yeast, in vivo)	2.00	1–0.02	1 antigenic

6	SSMKLSFRARAYGFRGPGPQL Mol wt 2327.00	Nontoxin	Nonhemolytic	Negative	15.74	−0.21 −0.49 −0.00	20 h (yeast, in vivo)	4.00	11.73	0 antigenic

7	TSAKFTEEKLPNYR Mol wt 1684.07	Nontoxin	Nonhemolytic	Negative	41.94	−0.37 −1.39 0.54	20 h (yeast, in vivo)	1.00	8.83	0 antigenic

8	ELQER Mol wt 673.79	Nontoxin	Nonhemolytic	Negative	111.72	−0.63 −2.24 1.48	30 min (yeast, in vivo)	−1.00	4.54	0 antigenic

9	IPALLKR Mol wt 810.15	Nontoxin	Nonhemolytic	Negative	66.41	−0.13 0.56 0.01	30 min (yeast, in vivo)	2.00	11.01	0 antigenic

10	ELQACQQVMDR Mol wt 1320.66	Nontoxin	Nonhemolytic	Negative	75.62	−0.32 −0.71 0.32	30 min (yeast, in vivo)	−1.00	4.38	0 antigenic

11	AQQLAAQLPAMCR Mol wt 1400.86	Nontoxin	Nonhemolytic	Negative	53.68	−0.12 0.20 −0.33	20 hours (yeast, in vivo)	1.00	8.60	0 antigenic

12	LEGGDALSASQ Mol wt 1047.24	Nontoxin	Nonhemolytic	Negative	37.82	−0.06 −0.15 0.20	3 min (yeast, in vivo)	−2.00	3.67	0 antigenic

13	FLPVLAGVLSRA Mol wt 1242.70	Nontoxin	Nonhemolytic	Negative	57.32	0.15 1.58 −0.72	3 min (yeast, in vivo)	1.00	10.11	0 antigenic

14	LLGTILGLLKGL Mol wt 1210.78	Nontoxin	Hemolytic	Negative	3.99	0.26 1.79 −0.83	3 min (yeast, in vivo)	1.00	9.11	0 antigenic

15	GLFDIIKKIAESF Mol wt 1480.96	Nontoxin	Hemolytic	Negative	20.48	0.04 0.67 −0.03	20 h (yeast, in vivo)	0.00	6.42	0 antigenic

16	RRWQWRMKKLG Mol wt 1545.05	Nontoxin	Nonhemolytic	Negative	105.52	−0.59 −1.94 0.48	2 min (yeast, in vivo)	5.00	12.31	0 antigenic

### 2.2. Energy Minimization of Predicted Structures

The proteins and peptides were structurally optimized using the YASARA server (https://www.yasara.org/minimizationserver.htm) to identify and correct errors in the selected models. The coordinates of the energy‐minimized three‐dimensional structures were saved in PDB format.

### 2.3. Protein Docking Study

Notably, we separately simulated the HE enzyme and peptides, and then used the resulting structures for a molecular docking study. To dock the 11 peptides to the HE protein, we utilized the HPEPDOCK server (http://huanglab.phys.hust.edu.cn/hpepdock/). The PDB structures of the HE protein and the peptides were uploaded to the server for docking. HPEPDOCK employs an automated blind docking protocol; therefore, user‐defined grid box coordinates and docking parameters are neither required nor provided by the server. After analyzing the docking results of all peptide–HE complexes, three peptides (Peptides 2, 12, and 16) were selected for MD simulations based on their interactions with the key amino acid residues of the HE active site, including Ser40, Asp326, and His329.

### 2.4. MD Simulations Study

MD simulations of all structures were performed using GROMACS Version 2016.4 with the CHARMM36 force field. All systems in this study were placed in a triclinic box, and TIP3p water molecules were used as the solvent. The HE protein, HE–Peptide 2, HE–Peptide 12, and HE–Peptide 16 complexes were solvated with 15,120, 19,043, 19,056, and 20,576 water molecules, respectively, and neutralized with 8 Na^+^, 8 Na^+^, 10 Na^+^, and 3 Na^+^ ions in water boxes of dimensions (10.7 Å × 7.8 Å × 6.1 Å), (8.3 Å × 8.2 Å × 9.4 Å), (8.3 Å × 8.2 Å × 9.4 Å), and (8.9 Å × 8.2 Å × 9.4 Å), respectively. The energy of each protein system was minimized using the steepest‐descent algorithm with an *emstep* of 0.01 and an *emtol* of 1000. Four systems were included in this study: the HE protein, the HE–Peptide 2 complex, the HE–Peptide 12 complex, and the HE–Peptide 16 complex. Each system was equilibrated with a 1 ns simulation under the NVT ensemble and a 2 ns simulation under the NPT ensemble to stabilize temperature (303.15 K) and pressure (1 bar), respectively. Initial velocities were generated using a V‐rescale thermostat based on Maxwell’s distribution. Position restraints were applied during both the NVT and NPT equilibration phases. The Verlet leapfrog integrator, with a time step of 2 fs, was used for all MD simulations. Periodic boundary conditions were applied in all directions. Long‐range electrostatic interactions were computed using the particle mesh Ewald (PME) method with an interpolation order of four. A cutoff distance of 1 nm was used for short‐range van der Waals and electrostatic interactions, and the LINCS algorithm was applied to constrain covalent bond lengths. For all systems, 200 ns production MD runs were performed. To investigate the stability and dynamic behavior of the proteins, the production MD trajectories of each system were analyzed using parameters such as root‐mean‐square deviation (RMSD), root‐mean‐square fluctuation (RMSF), radius of gyration (Rg), number of hydrogen bonds, and solvent‐accessible surface area (SASA). The secondary structures of the proteins were evaluated using the DSSP program. To enhance the reliability of the MD analyses, all simulations were conducted twice under the same conditions. The reported values for RMSD, RMSF, Rg, SASA, and hydrogen‐bond analyses represent averages from both simulation runs. It is important to note that the individual data from the first and second simulations are not shown; only the average values are reported. The PDBSUM web server (http://www.ebi.ac.uk/thornton-srv/databases/pdbsum/Generate.html) determined the amino acids involved in the interaction of two proteins.

## 3. Results

### 3.1. Molecular Docking Analysis

Protein–peptide docking is a molecular modeling approach that employs computational algorithms and techniques to predict how the two molecules interact and orient themselves during complex formation. The results of the eleven peptide–HE complexes—including the lowest energy of each model and the corresponding amino acid residues—are presented in Table S1 and Figure S1 of the Supporting Information file. Notably, the selection of the three peptides from the 11 candidates was based on their local interactions. Peptides 2 and 12 interact with amino acid residues located within the binding site of the HE protein, whereas Peptide 16 interacts with residues situated in the esterase domain of the HE protein [7].

#### 3.1.1. Results of the Molecular Docking Study on the Interaction Between Peptide (2, 12, 16)–HE Protein Complexes.

Molecular docking simulations and structural analyses were conducted using the PDBSUM server to precisely identify the binding mode and nature of molecular interactions between the candidate peptides (Peptides 2, 12, and 16) and the HE protein. The interaction diagrams, which include the key amino acid residues and types of intermolecular forces, are detailed in Figure S1 of the Supporting Information file; also, Table 3 shows the results for three selected peptides (Peptides 2, 12, and 16).

**TABLE 3:** Molecular docking results for the three peptides selected for molecular dynamics simulations, including docking scores and interactions with key amino acid residues of the HE protein.

Number peptide	Amino acid residues of HE involved in the interaction with peptide	Amino acid residues of peptides involved in the interaction with HE	Docking score
2	Tyr174\Arg177\Asn180\Phe181\Lys210 Phe211\Leu212\Tyr217\Glu265\Leu266 Leu267	Phe5\Asn6\Gly8\Ala9\ Asn11\Arg12	−171.57

12	Tyr184\Tyr185\Tyr186\Lys187\Phe211\ Ser213\Lys216\Tyr217\Tyr218\Asp219\ Asp220\Asn264	Glu2\Gly3\Gly4\Ala6\ Leu7\Ser10\Gln11	−142.59

16	Ser40\Arg41\Hsd46\Ser56\Tyr57\ Asp59\Tyr100\Arg292\Trp293\Asn295	Arg2\Trp3\Gln4\Arg6\ Met7\Lys8\Leu10\Gly11	−264.76

The PDBSUM analysis of Peptide 2 (Figure S1A) confirmed its binding to the target protein. The schematic illustrates that 11 amino acid residues from HE participate in the interaction with Peptide 2. The key residues in HE include Tyr174, Arg177, Asn180, Phe181, Lys210, Phe211, Leu212, Tyr217, Glu265, Leu266, and Leu267. This complex is stabilized through 81 nonbonded contacts and four hydrogen bonds.

According to the PDBSUM analysis of Peptide 12 (Figure S1B), a distinct binding profile was observed for the HE protein. As shown in the schematic diagram, 14 amino acid residues from HE interacted with Peptide 12. The critical HE residues involved in this interaction were Tyr184, Tyr185, Tyr186, Lys187, Phe211, Ser213, Asn214, Lys216, Tyr217, Tyr218, Asp219, Asp220, Tyr223, and Asn264. This complex was stabilized by 106 nonbonded contacts, eight hydrogen bonds, and one hydrophobic interaction.

Based on PDBSUM analysis (Figure S1C), Peptide 16 exhibited a distinct interaction pattern with the HE protein. The schematic diagram showed the involvement of 14 HE residues in the binding interface with Peptide 16. The key HE residues involved in this interaction were Ser40, Arg41, His46, Ser56, Tyr57, Asp59, Tyr100, Ser291, Arg292, Trp293, Asn295, Val324, Tyr325, and Phe334. The interaction is primarily stabilized by 138 nonbonded contacts representing hydrophobic and van der Waals interactions along with two hydrogen bonds.

Furthermore, the binding energy scores for Peptides 2, 12, and 16 were −171.57, −142.59, and −264.76, respectively.

### 3.2. MD Simulation Analysis

The MD simulation method was employed to monitor the stability and dynamic behavior of HE in the free state and in the vicinity of three selected Peptides (2, 12, 16) to determine whether these peptides can bind to HE and inhibit it.

#### 3.2.1. RMSD Analysis

**FIGURE 1:**
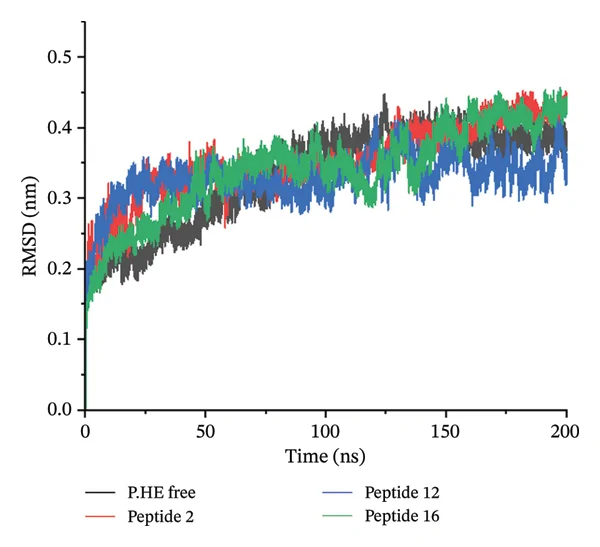
Root‐mean‐square‐deviation (RMSD) profiles of the free HE protein and its complexes with Peptides 2, 12, and 16. The curves represent the average values obtained from the first and second simulation runs. Black color: hemagglutinin esterase, red color: HE–Peptide 2 complex, blue color: HE–Peptide 12 complex and green color: HE–Peptide 16 complex.

RMSD is a widely used parameter in MD simulations to assess the overall structural stability of proteins and protein–ligand complexes by quantifying the deviation of atomic positions from a reference structure over time [27]. Lower, more stable RMSD values generally indicate structural convergence, whereas larger fluctuations reflect greater conformational flexibility. The structural stability of the free HE protein and its complexes with Peptides 2, 12, and 16 was evaluated over 200 ns of MD simulations (Figure 1). Following the initial equilibration phase, all systems exhibited relatively stable RMSD profiles throughout the remainder of the simulation. Among the peptide‐bound complexes, the HE protein–Peptide 12 complex displayed the lowest average RMSD values, whereas the HE–Peptide 2 and HE–Peptide 16 complexes showed slightly higher structural deviations during the simulation. These observations suggest that peptide binding did not compromise the overall structural stability of the HE protein under the simulated conditions.

#### 3.2.2. RMSF Analysis

**FIGURE 2:**
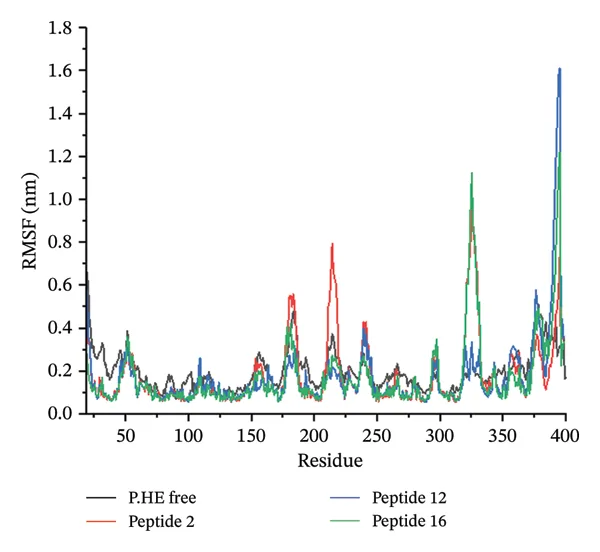
Root‐mean‐square‐fluctuation (RMSF) profiles of the free HE protein and its complexes with Peptides 2, 12, and 16. The curves represent the average values obtained from the first and second simulation runs. Black color: hemagglutinin esterase, red color: HE–Peptide 2 complex, blue color: HE–Peptide 12 complex, and green color: HE–Peptide 16 complex.

RMSF is commonly used to assess the flexibility of individual amino acid residues during MD simulations by measuring their positional fluctuations relative to their time‐averaged coordinates [27]. Regions with higher RMSF values generally correspond to flexible loops or terminal regions, whereas lower values indicate more rigid structural elements. The residue‐level flexibility of the free HE protein and its complexes with Peptides 2, 12, and 16 were evaluated using the average RMSF values obtained from the first and second MD simulations (Figure 2).

Overall, the RMSF profiles of the peptide‐bound complexes were generally comparable to those of the free HE protein, with localized differences observed in specific residue regions. The HE–Peptide 2 complex exhibited relatively higher fluctuations in several regions, particularly around residues 175–185, 205–220, 230–245, 320–335, and the C‐terminal region. The HE protein–Peptide 12 and HE protein–Peptide 16 complexes displayed RMSF profiles similar to that of the free protein across most residues, although increased flexibility was observed in the C‐terminal region.

Overall, peptide binding induced localized changes in residue flexibility without substantially altering the global fluctuation profile of the HE protein during the simulation.

#### 3.2.3. Rg Analysis

**FIGURE 3:**
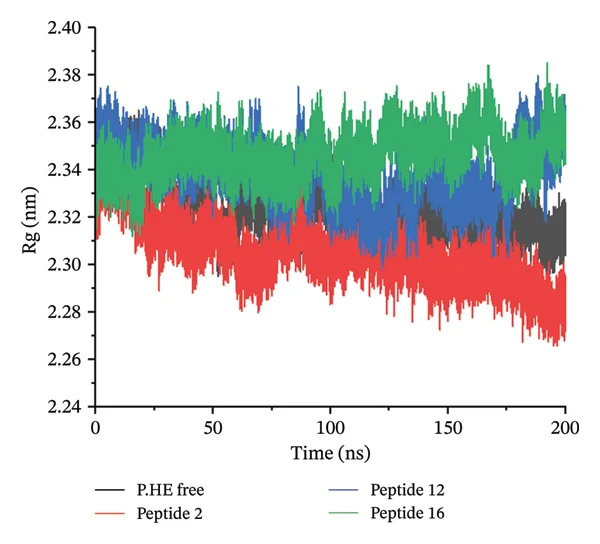
Radius of gyration (Rg) profiles of the free HE protein and its complexes with Peptides 2, 12, and 16. The curves represent the average values obtained from the first and second simulation runs. Black color: hemagglutinin esterase, red color: HE–Peptide 2 complex, blue color: HE–Peptide 12 complex, and green color: HE–Peptide 16 complex.

The Rg reflects the compactness of a molecular system and is calculated as the root mean square distance of atoms from their center of mass [27]. Changes in Rg during MD simulations may reflect alterations in the overall compactness of a protein structure. The Rg was calculated to assess the structural compactness of the free HE protein and its peptide‐bound complexes, using average values from the first and second MD simulations (Figure 3). The free HE protein exhibited a relatively stable Rg value of approximately 2.31 nm throughout the simulation. The HE–Peptide 2 complex showed a slightly lower average Rg value (approximately 2.28 nm), indicating a modest increase in structural compactness relative to the free protein. In contrast, the HE–Peptide 12 complex exhibited a slightly higher average Rg value (approximately 2.35 nm), suggesting a modest reduction in structural compactness. The HE protein–Peptide 16 complex displayed an average Rg value comparable to that of the free HE protein (approximately 2.34 nm). Overall, the Rg values remained relatively stable throughout the simulations, suggesting that peptide binding did not significantly alter the global compactness of the HE protein under the simulated conditions.

#### 3.2.4. Hydrogen Bond Analysis

**FIGURE 4:**
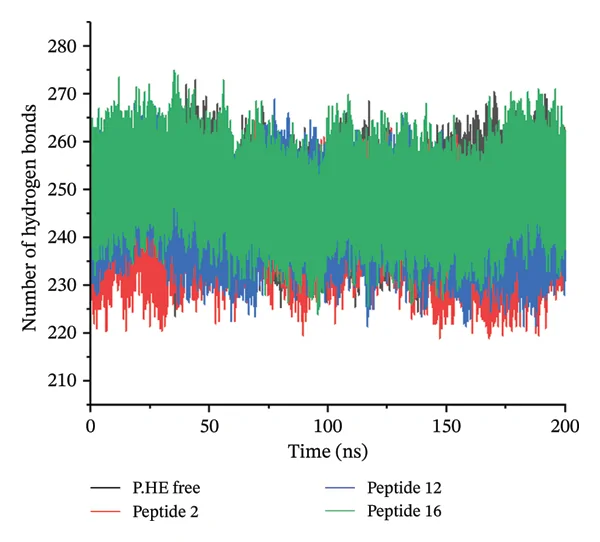
The number of intra‐molecular H‐bond profiles of the free HE protein and its complexes with Peptides 2, 12, and 16. The curves represent the average values obtained from the first and second simulation runs. Black color: hemagglutinin esterase, red color: HE–Peptide 2 complex, blue color: HE–Peptide 12 complex and green color: HE–Peptide 16 complex.

**FIGURE 5:**
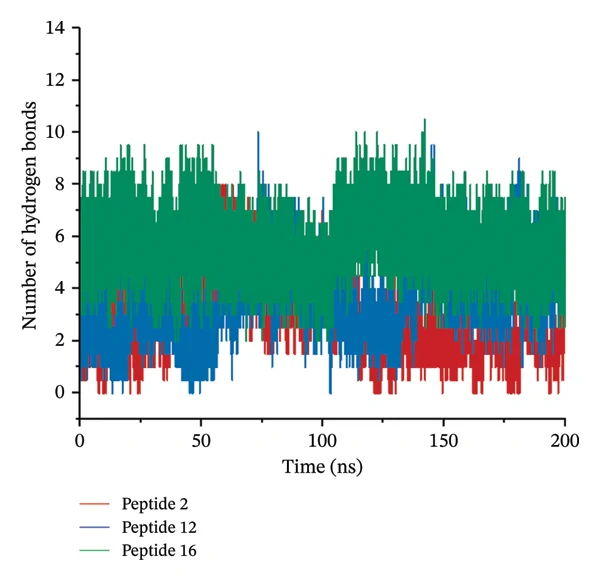
The number of intermolecular H‐bond profiles of the free HE protein and its complexes with Peptides 2, 12, and 16 The curves represent the average values obtained from the first and second simulation runs. Red color: HE–Peptide 2 complex, blue color: HE–Peptide 12 complex, and green color: HE–Peptide 16 complex.

Hydrogen‐bond analysis was performed to assess the stability of the HE protein and protein–peptide interactions during MD simulations. The average numbers of intra‐ and intermolecular hydrogen bonds were calculated using the average values obtained from the first and second MD simulations (Table 4; Figures 4 and 5).

**TABLE 4:** Intra‐ and intermolecular hydrogen bond analysis of the HE protein and its complexes with the three selected peptides during molecular dynamics simulations.

Compound name	Intra H‐bond	Inter H‐bond
Hemagglutinin–esterase	245.932	—
HE–Peptide 2	234.897	3.867
HE–Peptide 12	242.034	5.935
HE–Peptide 16	245.708	5.290

The free HE protein maintained an average of 247.776 intramolecular hydrogen bonds, indicating preservation of its internal hydrogen‐bonding network throughout the simulations. Similarly, the HE–Peptide 2, HE–Peptide 12, and HE–Peptide 16 complexes maintained average intramolecular hydrogen bond counts of 242.775, 245.594, and 256.340, respectively, suggesting that peptide binding did not markedly alter the internal hydrogen‐bonding network of the HE protein.

Analysis of intermolecular hydrogen bonds showed that Peptide 2 formed an average of 2.172 hydrogen bonds with the HE protein, whereas Peptides 12 and 16 formed averages of 2.863 and 5.960 hydrogen bonds, respectively. These findings suggest that Peptides 12 and 16 established more frequent protein–peptide hydrogen‐bond interactions than Peptide 2 during the simulations.

#### 3.2.5. Secondary Structure Analysis

To evaluate the structural effects of peptide binding on the HE protein, DSSP‐based secondary structure analyses were conducted on the free protein and its complexes with three peptide ligands: Peptide 2, Peptide 12, and Peptide 16. These analyses revealed that the overall secondary‐structure profile of HE is largely conserved across all states, indicating no major disorder in its global fold upon peptide interaction. Structural elements were listed under coil, *β*‐sheet, *β*‐bridge, bend, turn, *α*‐helix, and 3_10_‐helix elements (Table 5).

**TABLE 5:** Secondary structure analysis of the hemagglutinin esterase protein and its complexes with Peptides 2, 12, and 16 during molecular dynamics simulations.

Compound name	Structure	Coil	Beta sheet	Beta bridge	Bend	Turn	Alpha helix	3_10_‐helix
Hemagglutinin–esterase (Free)	0.46	0.36	0.25	0.02	0.17	0.08	0.12	0.01
HE–Peptide 2	0.44	0.37	0.22	0.02	0.18	0.08	0.11	0.01
HE–Peptide 12	0.47	0.33	0.25	0.03	0.19	0.09	0.11	0.01
HE–Peptide 16	0.44	0.36	0.23	0.03	0.18	0.08	0.11	0.02

The free HE protein exhibited a total structured content of 46%, with *β*‐sheets (25%) and coils (36%) dominating its secondary structure landscape. The *α*‐helix contribution was modest (12%), with minor contributions from bends (17%), turns (8%), *β*‐bridges (2%), and 3_10_‐helices (1%). There were only minor variations observed in secondary structure ratios upon binding of peptides, indicating that HE’s overall fold remained relatively conserved across complexes. Peptide 16 and Peptide 2 binding decreased the overall secondary‐structure component to 44%, whereas Peptide 12 binding increased it slightly to 47%. These changes potentially reflect peptide‐specific stabilization or local reorganization. Interestingly, binding with Peptide 12 slightly decreased the *β*‐sheet structure (from 25% to 23%) but modestly increased *β*‐bridges (from 2% to 3%), bends (from 19%), and turns (from 9%), suggesting localized motion or looping. Structure features of Peptide 16 were similar but exhibited a modest increase in 3_10_‐helix structure (2%), which could represent the incorporation of short helices near the binding interface.

In conclusion, these results suggest that peptide binding does not substantially restructure HE’s secondary structure but does induce slight local changes, most prominent in nonregular secondary‐structure elements (i.e., bends and bridges), that are perhaps functionally relevant to molecular recognition and complex assembly.

#### 3.2.6. SASA Analysis

**FIGURE 6:**
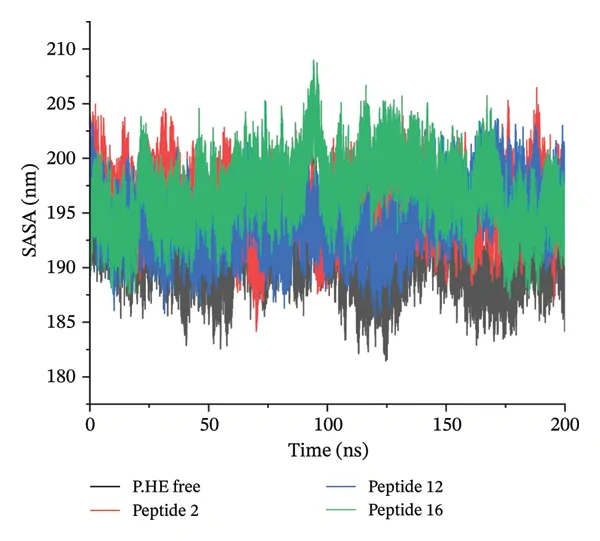
Solvent‐accessible surface area (SASA) profiles of the free HE protein and its complexes with Peptides 2, 12, and 16. The curves represent the average values obtained from the first and second simulation runs. Black color: hemagglutinin esterase, red color: HE–Peptide 2 complex, blue color: HE–Peptide 12 complex, and green color: HE–Peptide 16 complex.

SASA measures the surface area of a biomolecule accessible to solvent molecules and is commonly used to assess changes in solvent exposure during MD simulations [28]. Variations in SASA may reflect changes in protein compactness and solvent accessibility following ligand binding. The SASA values of the free HE protein and its peptide‐bound complexes were analyzed using the average values obtained from the first and second MD simulations (Figure 6).

The free HE protein exhibited lower SASA values than the peptide‐bound complexes throughout most of the simulation, indicating a smaller SASA. Following peptide binding, all three complexes displayed slightly higher SASA values with comparable fluctuation patterns. During the later stages of the simulations, the SASA values of the HE protein–Peptide 2, HE protein–Peptide 12, and HE protein–Peptide 16 complexes converged and remained within a similar range, suggesting that peptide binding resulted in comparable solvent accessibility among the three complexes under the simulated conditions.

#### 3.2.7. Minimum Distance Between Protein and Peptide

**FIGURE 7:**
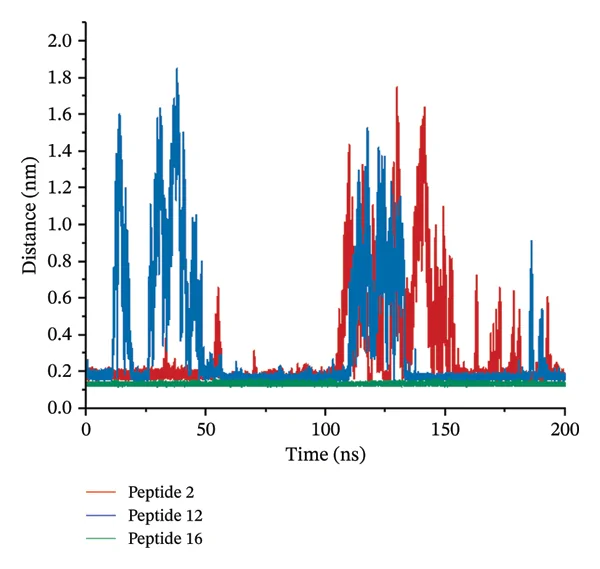
Distance plots between hemagglutinin esterase protein and Peptides 2, 12, and 16. The curves represent the average values obtained from the first and second simulation runs. Red color: HE–Peptide 2 complex, green color: HE–Peptide 12 complex, and blue color: HE–Peptide 16 complex.

Minimum distance analysis calculates the shortest distance between atoms or molecular surfaces and is often used to track the proximity of binding partners during simulations [29]. In this study, minimum‐distance analysis was performed to assess the closest distance between the HE protein and each peptide throughout the MD simulations. Lower minimum‐distance values indicate persistent molecular contact and stable binding. The results presented in (Figure 7) represent the average of two simulation runs. Among the three complexes, the HE–Peptide 16 complex exhibited the lowest and most stable minimum distance throughout the simulation, remaining close to 0.15 nm with only minor fluctuations. In contrast, the HE protein–Peptide 2 and HE protein–Peptide 12 complexes showed transient increases in the minimum distance during specific time intervals, indicating temporary fluctuations in protein–peptide contacts. However, in both systems, the minimum distance returned to values below approximately 0.3 nm after these fluctuations, suggesting that the peptides remained associated with the HE protein and no stable dissociation occurred during the 200 ns simulation. Overall, the minimum‐distance analysis indicates that Peptide 16 (bLFcin *f*) maintained the most persistent interaction with the HE protein. In contrast, Peptides 2 and 12 exhibited greater fluctuations in contacts while remaining bound throughout the simulation.

#### 3.2.8. Results of the MD Simulation Study on the Interaction Between Peptide (2, 12, 16)–HE Protein Complexes

**FIGURE 8:**
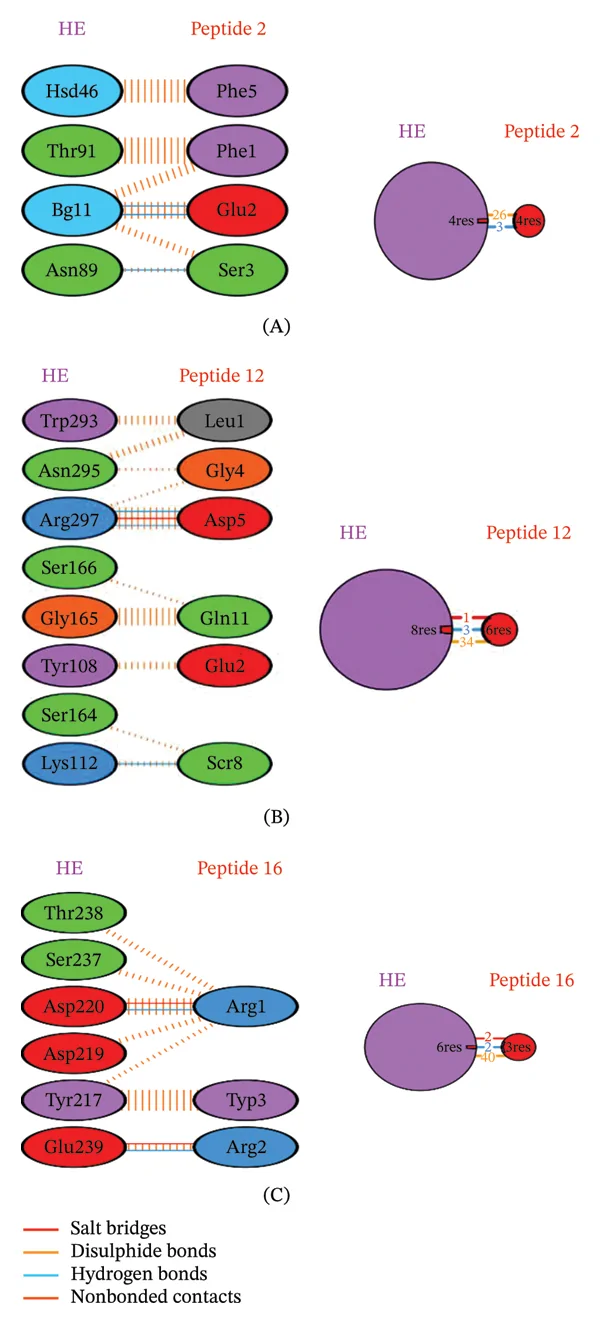
Molecular dynamics simulation results for the HE protein in complex with Peptides 2 (A), 12 (B), and 16 (C). The number of H‐bond lines between any two residues indicates the number of potential hydrogen bonds between them. For nonbonded contacts, which can be plentiful, the width of the striped line is proportional to the number of atomic contacts. Residue colors: positive (H, K, R) is shown blue; negative (D, E) is shown red; neutral (N, Q) is shown green; aliphatic (A, V, L) is shown gray; aromatic (W) is shown violet; pro and gly are shown beige.

Following MD simulation, the HE–Peptide 2 complex exhibited interactions involving four residues of the HE protein (Bgl1^1^, His46, Asn89, and Thr91) with four residues of Peptide 2 through 26 nonbonded contacts and three hydrogen bonds (Figure 8A). In the HE–Peptide 12 complex, eight residues of HE (Tyr108, Lys112, Ser164, Gly165, Ser166, Trp293, Asn295, and Arg297) interacted with six residues of Peptide 12, forming stable interactions, including 34 nonbonded contacts, three hydrogen bonds, and one specific salt bridge interaction (Figure 8B). In the HE–Peptide 16 complex, six residues of HE (Tyr217, Asp219, Asp220, Ser237, Thr238, and Glu239) maintained stable interactions with three residues of Peptide 16 via 40 nonbonded contacts, two hydrogen bonds, and two specific salt‐bridge interactions (Figure 8C). These results indicate that MD simulations facilitated the refinement of the binding interfaces and contributed to the identification of more stable interactions.

## 4. Discussion

Despite the widespread use of vaccination to reduce the incidence of BCoV infection, effective antiviral therapies for infected animals remain limited. Consequently, considerable efforts have been directed toward developing novel antiviral agents in veterinary medicine. Among these, bovine interferon alpha (BoIFN‐*α*) has attracted significant attention because of its potent antiviral and immunomodulatory activities. BoIFN‐*α* exerts its antiviral effects by inducing interferon‐stimulated genes (ISGs), inhibiting viral replication, and enhancing both innate and adaptive immune responses. Recent advances have demonstrated its potential for the prevention and treatment of several bovine viral diseases. However, challenges related to production costs, protein stability, and optimal delivery methods still limit its widespread clinical application [30–32]. In addition to interferon‐based therapies, passive immunotherapy using virus‐specific antibodies, hyperimmune colostrum, and host‐directed antiviral approaches has also been investigated as complementary strategies for controlling enteric and respiratory viral infections in cattle [33, 34].

In recent years, AVPs have emerged as a promising class of antiviral agents because of their high specificity, low cytotoxicity, and ability to target multiple stages of the viral life cycle. Unlike many conventional antiviral drugs that inhibit intracellular viral replication, AVPs frequently prevent viral attachment, receptor recognition, membrane fusion, or viral entry into host cells [35, 36]. These properties make peptide‐based therapeutics particularly attractive for coronaviruses, whose infection is initiated through interactions between viral surface proteins and host‐cell receptors. Although several antiviral strategies have been investigated in cattle, including interferon‐based therapies and passive immunization approaches, peptide‐based antivirals remain comparatively less explored against BCoVs.

In this study, to identify and evaluate the binding potential of three selected peptides (Peptide 2, Peptide 12, and Peptide 16) to the HE protein of BCoV and to assess the stability and dynamics of the resulting complexes, a combined approach of molecular docking and 200 ns MD simulations was employed. The HE protein plays a critical role in host cell attachment and viral entry in certain BCoV strains [7]. The results provide deeper insights into the molecular interactions and structural characteristics of these complexes, which could be instrumental in the development of novel antiviral agents.

RMSD, RMSF, Rg, hydrogen‐bond, and minimum‐distance analyses collectively indicated that all three peptide–HE protein complexes remained structurally stable during the simulations. Among them, the HE–Peptide 12 complex exhibited the lowest overall RMSD values, whereas Peptide 16 maintained the highest number of intermolecular hydrogen bonds and the most persistent contacts with the HE protein. These findings suggest that the three peptides interact differently with HE, with Peptide 16 showing particularly stable intermolecular interactions. The HE protein in coronaviruses, including BCoV, is involved not only in the initial binding to sialic acid receptors on host cell surfaces but also functions enzymatically as a RDE through its esterase activity. This enzymatic function prevents virion aggregation and facilitates viral release from infected cells [37]. A study by Schultze B et al. [38] demonstrated that BCoV uses N‐acetyl‐9‐O‐acetylneuraminic acid as a key receptor. Therefore, any interference with HE binding to this form of sialic acid or with its esterase activity may exert significant antiviral effects.

Based on the combined molecular docking and MD analyses, Peptide 16 exhibited persistent interactions with several residues located in the receptor‐binding region of the HE protein and maintained stable contacts throughout the simulations. These observations suggest that Peptide 16 may represent a promising candidate for further experimental evaluation. During the simulation, Peptide 16 maintained stable contact with key receptor‐binding site residues, including Tyr217, Asp219, Asp220, Ser237, Thr238, and Glu239. This interaction pattern suggests that Peptide 16 can occupy the enzyme’s receptor‐binding region and may interfere with receptor recognition. In contrast, Peptide 12 was localized around the receptor‐binding site without direct contact with the key amino acid residues of the receptor‐binding site. Peptide 2 mostly remained on the protein surface, with limited interactions with HE protein functional residues. Therefore, Peptide 16 may be a candidate for developing HE‐targeting inhibitors, although experimental validation is required to confirm its antiviral activity. It is plausible that Peptide 16 may interfere with HE protein function through steric hindrance at the receptor‐binding region. Because Peptide 16 interacted with residues located within the receptor‐binding region of HE, it may interfere with the interaction between HE and N‐acetyl‐9‐O‐acetylneuraminic acid. However, this hypothesis requires experimental confirmation [39].

The changes observed in the RMSF profile of the HE protein upon peptide binding reflect the peptide’s influence on the protein’s internal dynamics. Reduced flexibility in specific HE regions may indicate stabilization of local conformations that could influence receptor binding or esterase‐related functions. For example, changes in the flexibility of loop regions near the esterase active site could influence the accessibility of the catalytic pocket to sialic acid substrates [40].

Notably, in 2011, Asoodeh et al. isolated Peptide 2 from lysozyme hydrolysate and investigated its inhibitory effect on ACE1. Asoodeh reported that Peptide 2 has a strong inhibitory effect on ACE1 [20]. In our study, this peptide also interacted with amino acids in the active site of the HE of BCoV. Also, in 2014, Asoodeh et al. obtained Peptide 12 from trypsin hydrolysis of wheat gluten protein using bioinformatics techniques and investigated its inhibitory effect against ACE. They reported that Peptide 12 has a strong inhibitory effect on ACE1 [25].

Peptide 16 (bLFcin *f*) is derived from bovine lactoferricin (LfcinB), a bioactive peptide generated by the enzymatic digestion of bovine lactoferrin, an iron‐binding glycoprotein abundantly present in milk and other body fluids. Lactoferrin and its derived peptides exhibit a wide range of biological activities, including antimicrobial, antiviral, immunomodulatory, and anti‐inflammatory effects [41]. For example, Ammendolia et al. demonstrated that lactoferrin‐derived peptides contribute to innate immune defense against several pathogens, including influenza virus, by interfering with viral infection and modulating host immune responses [42]. In addition to their antiviral activity, lactoferricin‐derived peptides have been reported to inhibit the growth of *Vibrio cholerae* and other *Vibrio* species through membrane disruption and bactericidal mechanisms [43]. These findings suggest that lactoferrin‐derived peptides possess broad‐spectrum biological activities and support the rationale for investigating Peptide 16 (bLFcin *f*) as a potential antiviral candidate against BCoV. In the present study, Peptide 16 exhibited the most favorable overall interaction profile with the HE protein and maintained stable interactions throughout the MD simulations. Although further experimental studies are required to confirm its antiviral efficacy, our computational findings indicate that this peptide represents a promising candidate for future investigation.

## 5. Conclusion

In summary, the present study provides a computational assessment of the interactions between selected bioactive peptides and the HE protein of BCoV. Although the molecular docking and MD results suggest that Peptide 16 maintains more persistent interactions with the receptor‐binding region of HE than the other peptides evaluated, these findings should be interpreted with caution, as they are based on computational simulations. Experimental validation, including binding assays, esterase inhibition studies, and antiviral assays in cell culture, will be necessary to confirm Peptide 16’s inhibitory activity against BCoV.

## Author Contributions

The project was designed and directed by Mohammadreza Nassiri. Mohammed Adnan Hashim Al‐battat developed the theory and performed the computations. Marzieh Gharouni guided the performance of computer calculations and data analysis. Ali Javadmanesh and Ahmad Asoodeh were consultants for the entire project. Mohammed Adnan Hashim Al‐battat and Marzieh Gharouni contributed to the original writing of the manuscript. All authors reviewed the manuscript.

## Funding

This is part of a doctoral dissertation and is funded by the researchers themselves.

## Disclosure

The corresponding authors affirm that this manuscript is an honest, accurate, and transparent account of the study being reported; that no important aspects of the study have been omitted; and that any discrepancies from the study as originally planned have been explained.

## Ethics Statement

This was a computational biology study; it did not require an ethical verification code.

## Conflicts of Interest

The authors declare no conflicts of interest.

## Endnotes

^1^Bg: 2‐acetamido‐2‐deoxy‐beta‐D‐glucopyranose.

## Supporting Information

Additional supporting information can be found online in the Supporting Information section.

## Supporting information


**Supporting Information** Supporting information includes figures and tables to support the “RESULTS” sections of this study. Table S1. Molecular docking results of all analyzed peptides with the HE protein, including docking scores and interacting amino acid residues. The three peptides selected for molecular dynamics simulations are highlighted in green based on their interactions with key amino acid residues of the HE protein. Figure S1: Molecular docking study results for the HE protein in complex with peptides 2 (A), 12 (B), and 16 (C) and its guide. The number of H‐bond lines between any two residues indicates the number of potential hydrogen bonds between them. For non‐bonded contacts, which can be plentiful, the width of the striped line is proportional to the number of atomic contacts. Residue colors: Positive (H, K, R) as shown blue; negative (D, E) as shown red; neutral (N, Q) as shown green; aliphatic (A, V, L) as shown gray; aromatic (W) as shown violet; Pro & Gly as shown beige.

## Data Availability

All the data are available from the corresponding authors.

## References

[1] Cascella M. , Rajnik M. , Aleem A. , Dulebohn S. C. , and Di Napoli R. , Features, Evaluation, and Treatment of Coronavirus (COVID-19), 2020, StatPearls [Internet]. 32150360

[2] Decaro N. and Lorusso A. , Novel Human Coronavirus (SARS-CoV-2): A Lesson from Animal Coronaviruses, Veterinary Microbiology. (2020) 244, 10.1016/j.vetmic.2020.108693. PMC7195271 32402329

[3] Workman A. M. , McDaneld T. G. , Harhay G. P. , Das S. , Loy J. D. , and Hause B. M. , Recent Emergence of Bovine Coronavirus Variants With Mutations in the Hemagglutinin-Esterase Receptor Binding Domain in US Cattle, Viruses. (2022) 14, no. 10, 10.3390/v14102125. PMC9607061 36298681

[4] Saif L. J. and Jung K. , Comparative Pathogenesis of Bovine and Porcine Respiratory Coronaviruses in the Animal Host Species and SARS-CoV-2 in Humans, Journal of Clinical Microbiology. (2020) 58, no. 8, 10–1128, 10.1128/jcm.01355-20. PMC7383540 32522830

[5] Griffin D. , Chengappa M. M. , Kuszak J. , and McVey D. S. , Bacterial Pathogens of the Bovine Respiratory Disease Complex, Veterinary Clinics of North America: Food Animal Practice. (2010) 26, no. 2, 381–394, 10.1016/j.cvfa.2010.04.004. 20619191

[6] Langereis M. A. , Zeng Q. , Gerwig G. J. et al., Structural Basis for Ligand and Substrate Recognition by Torovirus Hemagglutinin Esterases, Proceedings of the National Academy of Sciences. (2009) 106, no. 37, 15897–15902, 10.1073/pnas.0904266106. PMC2747215 19721004

[7] Zeng Q. , Langereis M. A. , van Vliet A. L. W. , Huizinga E. G. , and de Groot R. J. , Structure of Coronavirus Hemagglutinin-Esterase Offers Insight Into Corona and Influenza Virus Evolution, Proceedings of the National Academy of Sciences. (July 2008) 105, no. 26, 9065–9069, http://www.pnas.org/content/105/26/9065.abstract, 10.1073/pnas.0800502105. PMC2449365 18550812

[8] Kienzle T. E. , Abraham S. , Hogue B. G. , and Brian D. A. , Structure and Orientation of Expressed Bovine Coronavirus Hemagglutinin-Esterase Protein, Journal of Virology. (1990) 64, no. 4, 1834–1838, 10.1128/jvi.64.4.1834-1838.1990. 2319653 PMC249325

[9] Zhang X. , Kousoulas K. G. , and Storz J. , The Hemagglutinin/Esterase Glycoprotein of Bovine Coronaviruses: Sequence and Functional Comparisons Between Virulent and Avirulent Strains, Virology. (1991) 185, no. 2, 847–852, 10.1016/0042-6822(91)90557-r. 1962455 PMC7131179

[10] Yoo D. and Pei Y. , Full-Length Genomic Sequence of Bovine Coronavirus (31Kb) Completion of the Open Reading Frame 1a/1b Sequences, The Nidoviruses: Coronaviruses and Arteriviruses, 2001, Springer, 73–76. 11774548

[11] Woo P. C. Y. , Lau S. K. P. , Huang Y. , and Yuen K.-Y. , Coronavirus Diversity, Phylogeny and Interspecies Jumping, Experimental Biology and Medicine. (2009) 234, no. 10, 1117–1127, 10.3181/0903-mr-94. 19546349

[12] Sánchez C. M. , Gebauer F. , Suñé C. , Mendez A. , Dopazo J. , and Enjuanes L. , Genetic Evolution and Tropism of Transmissible Gastroenteritis Coronaviruses, Virology. (1992) 190, no. 1, 92–105, 10.1016/0042-6822(92)91195-z. 1326823 PMC7131265

[13] La Manna S. , Di Natale C. , Florio D. , and Marasco D. , Peptides as Therapeutic Agents for Inflammatory-Related Diseases, International Journal of Molecular Sciences. (2018) 19, no. 9, 10.3390/ijms19092714. PMC6163503 30208640

[14] Xia S. , Yan L. , Xu W. et al., A pan-coronavirus Fusion Inhibitor Targeting the HR1 Domain of Human Coronavirus Spike, Science Advances. (2019) 5, no. 4, 10.1126/sciadv.aav4580. PMC6457931 30989115

[15] Tonk M. , Růžek D. , and Vilcinskas A. , Compelling Evidence for the Activity of Antiviral Peptides Against SARS-CoV-2, Viruses. (2021) 13, no. 5, 10.3390/v13050912. PMC8156787 34069206

[16] Berendsen H. J. C. , van der Spoel D. , and van Drunen R. , GROMACS: A message-passing Parallel Molecular Dynamics Implementation, Computer Physics Communications. (1995) 91, no. 1–3, 43–56, 10.1016/0010-4655(95)00042-e.

[17] Van Der Spoel D. , Lindahl E. , Hess B. , Groenhof G. , Mark A. E. , and Berendsen H. J. C. , GROMACS: Fast, Flexible, and Free, Journal of Computational Chemistry. (2005) 26, no. 16, 1701–1718, 10.1002/jcc.20291. 16211538

[18] Berman H. M. , Westbrook J. , Feng Z. et al., The Protein Data Bank, Nucleic Acids Research. (2000) 28, no. 1, 235–242. 10592235 10.1093/nar/28.1.235 PMC102472

[19] Hospital A. , Goñi J. R. , Orozco M. , and Gelpí J. L. , Molecular Dynamics Simulations: Advances and Applications, Adv Appl Bioinformatics Chem AABC. (2015) 8, 10.2147/aabc.s70333. PMC4655909 26604800

[20] Asoodeh A. , Yazdi M. M. , and Chamani J. , Purification and Characterisation of Angiotensin I Converting Enzyme Inhibitory Peptides From Lysozyme Hydrolysates, Food Chemistry. (2012) 131, no. 1, 291–295.

[21] Memarpoor-Yazdi M. , Asoodeh A. , and Chamani J. , Structure and ACE-Inhibitory Activity of Peptides Derived from Hen Egg White Lysozyme, International Journal of Peptide Research and Therapeutics. (2012) 18, no. 4, 353–360, 10.1007/s10989-012-9311-2.

[22] Ghavami S. , Asoodeh A. , Klonisch T. et al., Brevinin‐2R1 Semi‐Selectively Kills Cancer Cells by a Distinct Mechanism, Which Involves the Lysosomal‐Mitochondrial Death Pathway, Journal of Cellular and Molecular Medicine. (2008) 12, no. 3, 1005–1022, 10.1111/j.1582-4934.2008.00129.x. 18494941 PMC4401144

[23] Asoodeh A. , Zardini H. Z. , and Chamani J. , Identification and Characterization of Two Novel Antimicrobial Peptides, Temporin‐Ra and Temporin‐Rb, from Skin Secretions of the Marsh Frog (*Rana ridibunda*), Journal of Peptide Science. (2012) 18, no. 1, 10–16, 10.1002/psc.1409. 21956830

[24] Mohseni S. , Emtenani S. , Emtenani S. , and Asoodeh A. , Antioxidant Properties of a Human Neuropeptide and Its Protective Effect on Free Radical‐Induced DNA Damage, Journal of Peptide Science. (2014) 20, no. 6, 429–437, 10.1002/psc.2634. 24723458

[25] Asoodeh A. , Haghighi L. , Chamani J. , Ansari-Ogholbeyk M. A. , Mojallal-Tabatabaei Z. , and Lagzian M. , Potential Angiotensin I Converting Enzyme Inhibitory Peptides From Gluten Hydrolysate: Biochemical Characterization and Molecular Docking Study, Journal of Cereal Science. (2014) 60, no. 1, 92–98, 10.1016/j.jcs.2014.01.019.

[26] Furlund C. B. , Ulleberg E. K. , Devold T. G. et al., Identification of Lactoferrin Peptides Generated by Digestion With Human Gastrointestinal Enzymes, Journal of Dairy Science. (2013) 96, no. 1, 75–88, 10.3168/jds.2012-5946. 23141828

[27] Ghahremanian S. , Rashidi M. M. , Raeisi K. , and Toghraie D. , Molecular Dynamics Simulation Approach for Discovering Potential Inhibitors Against SARS-CoV-2: A Structural Review, Journal of Molecular Liquids. (2022) 354, 10.1016/j.molliq.2022.118901. PMC8916543 35309259

[28] Durham E. , Dorr B. , Woetzel N. , Staritzbichler R. , and Meiler J. , Solvent Accessible Surface Area Approximations for Rapid and Accurate Protein Structure Prediction, Journal of Molecular Modeling. (2009) 15, no. 9, 1093–1108, 10.1007/s00894-009-0454-9. 19234730 PMC2712621

[29] Bjelkmar P. , Larsson P. , Cuendet M. A. , Hess B. , and Lindahl E. , Implementation of the CHARMM Force Field in GROMACS: Analysis of Protein Stability Effects from Correction Maps, Virtual Interaction Sites, and Water Models, Journal of Chemical Theory and Computation. (2010) 6, no. 2, 459–466, 10.1021/ct900549r. 26617301

[30] Hai-Yang Y. U. , Dong-Mei G. A. O. , and Jun Z. , Research Progress of Bovine Interferon Alpha (BoIFN-α) in Veterinary Science: A Review, Kafkas Üniversitesi Vet Fakültesi Derg.(2026) 32, no. 1.

[31] Ce S. , Antiviral Actions of Interferons, Clinical Microbiology Reviews. (2001) 14, 778–809. 11585785 10.1128/CMR.14.4.778-809.2001 PMC89003

[32] Fensterl V. and Sen G. C. , Interferons and Viral Infections, BioFactors. (2009) 35, no. 1, 14–20, 10.1002/biof.6. 19319841

[33] Saif L. J. , Bovine Respiratory Coronavirus, Veterinary Clinics of North America: Food Animal Practice. (2010) 26, no. 2, 349–364, 10.1016/j.cvfa.2010.04.005. 20619189 PMC4094360

[34] Cho Y. and Yoon K.-J. , An Overview of Calf Diarrhea-Infectious Etiology, Diagnosis, and Intervention, Journal of Veterinary Science. (2014) 15, no. 1, 10.4142/jvs.2014.15.1.1. PMC3973752 24378583

[35] Vilas Boas L. C. P. , Campos M. L. , Berlanda R. L. A. , de Carvalho Neves N. , and Franco O. L. , Antiviral Peptides as Promising Therapeutic Drugs, Cellular and Molecular Life Sciences. (2019) 76, no. 18, 3525–3542, 10.1007/s00018-019-03138-w. 31101936 PMC7079787

[36] Mahendran A. S. K. , Lim Y. S. , Fang C.-M. , Loh H.-S. , and Le C. F. , The Potential of Antiviral Peptides as COVID-19 Therapeutics, Frontiers in Pharmacology. (2020) 11, 10.3389/fphar.2020.575444. PMC7522797 33041819

[37] Singhal T. , A Review of Coronavirus disease-2019 (COVID-19), Indian Journal of Pediatrics. (2020) 87, no. 4, 281–286, 10.1007/s12098-020-03263-6. 32166607 PMC7090728

[38] Schultze B. and Herrler G. , Bovine Coronavirus Uses N-acetyl-9-O-Acetylneuraminic Acid as a Receptor Determinant to Initiate the Infection of Cultured Cells, Journal of General Virology. (1992) 73, no. 4, 901–906, 10.1099/0022-1317-73-4-901. 1321878

[39] Wasik B. R. , Barnard K. N. , Ossiboff R. J. et al., Distribution of O-Acetylated Sialic Acids Among Target Host Tissues for Influenza Virus, mSphere. (2017) 2, no. 5, 10–1128, 10.1128/msphere.00379-16. PMC5588038 28904995

[40] Ahmed T. A. E. and Hammami R. , Recent Insights Into Structure–Function Relationships of Antimicrobial Peptides, Journal of Food Biochemistry. (2019) 43, no. 1, 10.1111/jfbc.12546. 31353490

[41] Wakabayashi H. , Yamauchi K. , and Takase M. , Lactoferrin Research, Technology and Applications, International Dairy Journal. (2006) 16, no. 11, 1241–1251, 10.1016/j.idairyj.2006.06.013.

[42] Ammendolia M. G. , Agamennone M. , Pietrantoni A. et al., Bovine Lactoferrin-Derived Peptides as Novel Broad-Spectrum Inhibitors of Influenza Virus, Pathogens and Global Health. (March 2012) 106, no. 1, 12–19, 10.1179/2047773212y.0000000004. 22595270 PMC4001507

[43] Acosta-Smith E. , Viveros-Jiménez K. , Canizalez-Román A. et al., Bovine Lactoferrin and Lactoferrin-Derived Peptides Inhibit the Growth of *Vibrio cholerae* and Other Vibrio Species, Front Microbiol [Internet]. (2018) 8–2017, https://www.frontiersin.org/journals/microbiology/articles/10.3389/fmicb.2017.02633. 29375503 10.3389/fmicb.2017.02633 PMC5768654

