# Supplementary material for: Evaluation of the Therapeutic Potential of Natural Peptides Against Hemagglutinin–Esterase of Bovine Coronavirus Based on Molecular Dynamics Simulation Studies
Source: Vet Med Int. 2026 Sep 15;2026:2269384. doi: 10.1155/vmi/2269384 (PMC13575882; doi:10.1155/vmi/2269384)
Supplement: Supplementary file 1 [file VMI-2026-2269384-s001.docx]

**Evaluation of the Therapeutic Potential of Natural Peptides Against Hemagglutinin-Esterase of Bovine Coronavirus Based on Molecular Dynamics Simulation Studies**

Mohammed Adnan Hashim Al-battat^1^, Marzieh Gharouni^2*^, Mohammadreza Nassiri^1,3*^, Ali Javadmanesh^1^, Ahmad Asoodeh^3,4^, Reza Tohidi^5^

*^1^*Department of Animal Science, Faculty of Agriculture, Ferdowsi University of Mashhad, Mashhad, Iran.

^2^ Metabolic Syndrome Research Center, Mashhad University of Medical Sciences, Mashhad, Iran.

^3^The Research Institute of Biotechnology, Ferdowsi University of Mashhad, Mashhad, Iran.

^4^Department of Chemistry, Faculty of Science, Ferdowsi University of Mashhad, Mashhad, Iran.

^5^Department of Animal Science, Faculty of Agriculture, University of Torbat-e Jam

Mohammed Adnan Hashim Al-battat: albattatmohammed551@gmail.com

Marzieh Gharouni: GharouniM4031@mums.ac.ir

Mohammadreza Nassiri: [nassiryr@um.ac.ir](mailto:nassiryr@um.ac.ir)

Ali Javadmanesh: javadmanesh@um.ac.ir

Ahmad Asoodeh: asoodeh@um.ac.ir

*Corresponding authors:

Mohammadreza Nassiri: nassiryr@um.ac.ir, ORICID ID: [0000-0001-7119-8155](https://orcid.org/0000-0001-7119-8155)

Marzieh Gharouni: GharouniM4031@mums.ac.ir, ORICID ID: 0000-0001-8214-4603

**Table S1.** Molecular docking results of all analyzed peptides with the HE protein, including docking scores and interacting amino acid residues. The three peptides selected for molecular dynamics simulations are highlighted in green based on their interactions with key amino acid residues of the HE protein.

| **Number peptide** | **Sequence of peptide** | **Amino acid residues of HE involved in the interaction with peptide** | **Amino acid residues of peptide involved in the interaction with HE** | **Docking Score** |
| --- | --- | --- | --- | --- |
| 1 | KAEDTQTLPFR | Gln132\Lys135\Asn136\Val139\Glu199\Val253\ Pro255\Arg280\Lys281\  Phe283\Tyr311\Tyr387\ Pro394 | Gln6\Thr7\Leu8\Pro9\Phe10\Arg11 | -181.89 |
| 2 | FESAFNTGATNR | Tyr174\Arg177\Asn180\  Phe181\Lys210\Phe211\  Leu212\Tyr217\Glu265\  Leu266\Leu267 | Phe5\Asn6\Gly8\Ala9\Asn11\Arg12 | -171.57 |
| 6 | SSMKLSFRARAYGFRGPGPQL | Leu128\The131\Gln132\Lys135\Asn136\Val139\Glu199\Asn236\Hsd250\Val253\Arg280\Phe283\  Pro310\Tyr311\Leu392\  Pro394 | Ser1\Ser2\Met3\Lys4\Leu5\Arg10\Ala11\  Tyr12\Phe14\Pro19 | -242.76 |
| 7 | TSAKFTEEKLPNYR | Gln132\Lys135\Val139\Arg280\Phe283\Pro310\Tyr311\lle384\Tyr387\  Leu392\Pro394 | Ser2\Ala3\Lys9\Leu10\Pro11\Asn12\Tyr13\  Arg14 | -191.67 |
| 8 | ELQER | Asp290\Asg292\Asn301\Ala304\Val305\Gln308\Phe314\Asn316\Thr318\  Thr319\Gln350 | Glu1\Leu2\Gln3\Glu4\Arg5 | -144.86 |
| 9 | IPALLKR | Bgl2\Gln132\Lys135\Asn136\Val253\Arg280\Lys281\Asp282\Phe283\  Gln308\Pro309\Pro310\  Tyr311\Tyr387\Leu392\  Val393\Pro394\Arg395 | IIe1\Pro2\Ala3\Leu4\  Leu5\Lys6\Arg7 | -157.78 |
| 10 | ELQACQQVMDR | Gln132\Lts135\Asn136\  Val139\Tyr140\Asp198\  Glu199\Val253\Pro255\  Arg280\Tyr311 | Ala4\Cys5\Val8\Met9\Asp10\Arg11 | -170.92 |
| 11 | AQQLAAQLPAMCR | Thr131\Gln132\Lys135\  Asn136\Val139\  Asn236\Tyr251\Leu252\Val253\Lys281\Asp282\  Phe283\Pro310\Tyr311\  Tyr387\Pro394 | Gln2\Gln3\Leu4\Gln7\Leu8\Met11\Cys12\  Arg13 | -206.50 |
| 12 | **LEGGDALSASQ** | Tyr184\Tyr185\Tyr186\  Lys187\Phe211\Ser213\  Lys216\Tyr217\Tyr218\  Asp219\Asp220\Asn264 | Glu2\Gly3\Gly4\Ala6\Leu7\Ser10\Gln11 | -142.59 |
| 13 | FLPVLAGVLSRA | Lys73\Phe87\Tyr88\  Asn89\Asn147\Val148\  Pro149\Asn170\Ala173\  Thr272\Lys273 | Phe1\Leu2\Pro3\Leu5\Leu9\Ser10\Arg11\  Ala12 | -196.35 |
| 16 | **RRWQWRMKKLG** | Ser40\Arg41\Hsd46\Ser56\Tyr57\Asp59\Tyr100\  Arg292\Trp293\Asn295 | Arg2\Trp3\Gln4\Arg6\Met7\Lys8\Leu10\  Gly11 | -264.76 |

**Fig. S1.** Molecular docking study results for the HE protein in complex with peptides 2 (A), 12 (B), and 16 (C) and its guide.

The number of H-bond lines between any two residues indicates the number of potential hydrogen bonds between them. For non-bonded contacts, which can be plentiful, the width of the striped line is proportional to the number of atomic contacts.

**Residue colors:** Positive (H, K, R) as shown blue; negative (D, E) as shown red; neutral (N, Q) as shown green; aliphatic (A, V, L) as shown gray; aromatic (W) as shown violet; Pro & Gly as shown beige.
